# Transplantation of human adipose stem cell-derived hepatocyte-like cells with restricted localization to liver using acellular amniotic membrane

**DOI:** 10.1186/s13287-015-0208-9

**Published:** 2015-11-05

**Authors:** Jie Yuan, Weihong Li, Jieqiong Huang, Xinyue Guo, Xueyang Li, Xin Lu, Xiaowu Huang, Haiyan Zhang

**Affiliations:** Department of Cell Biology, Municipal Laboratory for Liver Protection and Regulation of Regeneration, Capital Medical University, No. 10, Xitoutiao, You An Men, Beijing, 100069 China; Fu Xing Hospital, Capital Medical University, No. 20, Fu xing men wai, Beijing, 100038 China

## Abstract

**Introduction:**

Adult stem cell-derived hepatocytes transplantation holds considerable promise for future clinical individualized therapy of liver failure or dysfunction. However, the low engraftment of the available hepatocytes in the liver disease microenvironment has been a major obstacle.

**Methods:**

Acellular human amniotic membrane was developed as a three-dimensional scaffold and combined with hepatocyte-like cells derived from human adipose stem cells to engineer a hepatic tissue graft that would allow hepatocyte engraftment in the liver effectively.

**Results:**

The hepatic tissue grafts maintained hepatocyte-specific gene expression and functionality *in vitro*. When transplanted into the surgical incision in livers for engraftment, the engineered hepatic grafts significantly decreased the degree of liver injury caused by a carbon tetrachloride treatment and generated cords that were similar to the ductal plates in the liver between the acellular human amniotic membrane and the liver of receipts at day 3 post-transplantation. The hepatic tissue grafts maintained the expression of human hepatocyte-specific markers albumin, hepatocyte nuclear factor 4α, and cytochrome P450 2B6 in the liver of receipts, and acquired human-specific drug metabolism ability at eight weeks post-transplantation.

**Conclusions:**

The acellular human amniotic membrane has the ability to maintain the functional phenotype of the hepatocyte-like cells derived from human adipose stem cells. Functional acellular human amniotic membrane-hepatocytes grafts integrated with the liver decreases the acute liver injury of mice. These engineered tissue constructs may support stem cell-based individualized therapy for liver disease and for bioartificial liver establishment.

**Electronic supplementary material:**

The online version of this article (doi:10.1186/s13287-015-0208-9) contains supplementary material, which is available to authorized users.

## Introduction

Hepatocyte transplantation, especially using hepatocytes derived from patient adipose stem cells (ASCs), might become safer and easier than whole organ transplantation to cure patients suffering from liver-based metabolic diseases or end-stage liver dysfunction [1–4]. The delivery of these cells and promotion of their efficient engraftment is a challenging task [5]. Placing healthy hepatocytes via intrahepatic injection, intrasplenic delivery, or portal vein infusion may hinder engraftment because most diseased livers have altered architectures due to fibrosis and cirrhosis [6]. Therefore, implantation or extrahepatic transplantation to provide an additional site of hepatic function represents a new approach for hepatocyte transplantation [7–9]. New developments in liver engineering technology have motivated research into the development of functional liver grafts that could be connected to a recipient’s system [10, 11].

An ideal scaffold with relevant aspects of the hepatic microarchitecture and extracellular matrix (ECM) components plays important roles in hepatocyte adhesion and differentiation, as well as in promoting tissue morphogenesis, to create implantable liver tissues or grafts [12–14]. Human amniotic membrane (HAM) has been widely used as a graft material for many surgical procedures and for tissue regeneration because this material is inexpensive and easily obtained and because its availability is virtually limitless [15]. Before the material is applied, the amniotic membrane (AM) donor is required to undergo a thorough health screening, and the membrane must undergo an established processing routine, which includes preservation, sterilization, and de-epithelialization [16]. ECM components of acellular human amniotic membrane (AHAM), such as collagen type I, collagen type IV, laminin, and fibronectin, which have biological properties similar to the hepatic ECM, make this membrane a potentially attractive grafting material to facilitate hepatocyte transfer [17, 18]. Recent findings indicate that HAM may reduce the severity of liver fibrosis in a bile duct ligation rat model [19, 20]. However, little is known about the possibility of AHAM as a scaffold for hepatocyte attachment, functional maintenance, and transplantation. The aims of this study were to determine the biocompatibility of AHAM with human adipose stem cell-derived hepatocyte-like cells (hASC-HLCs) [21] and to assess the ability of AHAM to maintain the function of hepatocytes *in vitro* and in a hepatic implant in a carbon tetrachloride (CCl4)-induced acute hepatic injury mouse model *in vivo*.

## Methods

### Preparation of the AHAM

HAM was obtained from a cesarean section operation with informed patient consent and under the approval of the Ethics Committee of Capital Medical University (Beijing, China). A maternal donor with no history of premature membrane rupture, endometritis, or meconium ileus was selected at a prenatal visit approximately 2 weeks before delivery and underwent a series of serological tests, including screens for HIV-1/2, hepatitis B, hepatitis C, human T-cell lymphotropic virus type, syphilis, cytomegalovirus, and tuberculosis. Repeat investigations were performed 6 months after delivery.

To prepare the AHAM, the HAM was peeled from the placenta, rinsed extensively in sterile phosphate-buffered saline (PBS) containing 200 U/mL penicillin, 200 ug/mL streptomycin, and cut into approximately 5 cm × 5 cm pieces, which were placed in dishes with the amniotic epithelial layer face up. The HAM pieces were incubated in 0.25 % trypsin with 0.38 % ethylenediamine tetraacetic acid (EDTA; Sigma-Aldrich, St. Louis, MO, USA) for 30 minutes twice at 37 °C, followed by rinsing in sterile PBS and dehydration in glycerol for 48 hours; the glycerol was changed every day. Next, the fresh AHAM pieces were stored at 4 °C for up to 2 weeks. For generating cryopreserved AHAM, the fresh AHAM pieces were placed in dishes with a 1:1 mixture of glycerol and 0.5 % chondroitin sulfate (Sigma-Aldrich) in MEM-NEAA (Gibco, Carlsbad, CA, USA) and stored at –80 °C for several months [16].

At 24 hours before examination or cell seeding, the fresh and cryopreserved AHAMs were rehydrated with sterile PBS, cut into approximately 1.5 cm × 1.5 cm pieces, which were placed into 24-well cell culture plates with the basement membrane face up, and cultured with MEM-NEAA (Gibco) medium for at least 12 hours.

### Cell culture and cell seeding

hASCs were cultured and differentiated to hepatocytes as described previously [21]. Once the hASC-HLCs were differentiated, these cells were harvested with 0.05 % trypsin–0.02 % EDTA solution and resuspended in hepatic differentiation medium at a density of 3 × 10^5^/ml. The primary human hepatocytes (ScienCell Research Laboratories, Carlsbad, CA, USA) were cultured on collagen type I-coated six-well plates (3 × 10^5^ cells/well) with hepatocyte medium (ScienCell Research Laboratories).

The rehydrated cryopreserved AHAM pieces were divided into two groups. In the two-dimensional (2D) group, the AHAM pieces were spread on culture plates and then air-dried for 2 hours before seeding the cells; in the three-dimensional (3D) group, the AHAM pieces were spread on culture plates and maintained in MEM-NEAA medium without air-drying before cell seeding. hASC-HLCs were seeded on 2D-AHAM and 3D-AHAM at a density of 1 × 10^5^/cm^2^, with hASC-HLCs plated on collagen type I-coated 24-well cell culture plates as the control.

### Real-time RT-PCR

Real-time RT-PCR was performed as described previously [21, 22]. Total cellular RNA was extracted from 3 × 10^5^ cells with the RNeasy Mini Kit (QIAGEN, Hilden, Germany) according to the manufacturer’s instructions. For PCR analysis, 1 μg RNA was reverse-transcribed to cDNA using Superscript III reverse transcriptase and random hexamer primers (Invitrogen, Carlsbad, CA, USA). Real-time PCR analysis was performed on an ABI Prism 7300 Sequence Detection System using the SYBR Green PCR Master Mix (Applied Biosystems, Foster City, CA, USA). The reaction consisted of 10 μl SYBR Green PCR Master Mix, 1 μl of a 5 μM mix of forward and reverse primers, 8 μl water, and 1 μl template cDNA in a total volume of 20 μl. Cycling was performed using the default conditions of the ABI 7300 SDS Software 1.3.1 (Applied Biosystems). The relative expression of each gene was normalized against 18S rRNA. Data are presented as the mean ± standard deviation (SD). The primers used are presented in Additional file 1.

### Histochemistry and immunofluorescence

The cells or tissue section were fixed with 4 % paraformaldehyde for 20 minutes at room temperature, followed by permeabilization with 0.3 % Triton X-100 in PBS for 5 minutes. The cells were rinsed and blocked with 20 % goat serum (ZSGB-BIO, Beijing, China) or 1 % gelatin (Sigma-Aldrich) for 60 minutes at room temperature. The cells were then incubated with the following antibodies against human antigens rabbit anti-albumin (ALB) at 1:200 (Sigma-Aldrich), rabbit anti-Cytochrome P450 (CYP) 2B6 at 1:50 (Santa Cruz Biotechnology, Dallas, TX, USA), rabbit anti-collagen I at 1:100, rabbit anti-fibronectin at 1:200, mouse anti-collagen IV at 1:100, mouse anti-laminin at 1:200 (ZSGB-BIO), mouse anti-multidrug resistance-related protein 2 (MRP2) at 1:100 (Santa Cruz), mouse anti-hepatocyte nuclear factor (HNF) 4α at 1:50 (Santa Cruz), and mouse anti-human nuclei at 1:1000 (Millipore, Darmstadt, Germany) or rat monoclonal antibody against mouse CD31 at 1:50 (Santa Cruz) at 4 °C overnight. A Vector® M.O.M.™ immunodetection kit (Vector Laboratories, Inc., Burlingame, CA, USA) was used according to the manufacturer’s protocol to detect the mouse primary monoclonal antibodies for HNF4α and human nuclei on mouse tissues after hASC-HLC–3D-AHAM transplantation. Following three 5-minute washes in PBS with gentle agitation, an Alexa Fluor-conjugated secondary antibody (1:500; Invitrogen) was added, and the samples were incubated for 1 hour at 37 °C. The nuclei were counterstained with 4′,6-diamidino-2-phenylindole (DAPI; Sigma-Aldrich). The stained cells or tissue sections were examined under a Leica TCS SP8 confocal microscope (Leica, Wetzlar, Germany). For detecting the expression of CD31, the tissue sections were examined with the PV-6004 Polink-1 HRP DAB detection system and ZLI-9017 DAB Kit (ZSGB-BIO). The tissue sections were examined under an Axio Imager A2 microscope (Zeiss, Oberkochen, Germany).

### Scanning electron microscopy

For scanning electron microscopy (SEM) analysis, the samples were fixed in 3 % glutaraldehyde in 0.1 M phosphate buffer, pH 7.2, for 120 minutes at 4 °C, postfixed in 1 % osmium tetroxide for 60 minutes at room temperature, and dehydrated in 50 %, 70 %, 80 %, 90 %, and 100 % ethanol for 10 minutes, respectively. The samples were then air dried, mounted, sputter coated with gold, and examined using a Hitachi S-4800 scanning electron microscope (Hitachi, Tokyo, Japan).

### Transmission electron microscopy

For the ultra-structural analysis, the differentiated cells were fixed in 2.5 % glutaraldehyde in 0.1 M phosphate buffer, pH 7.2, for 120 minutes at 4 °C and postfixed in 1 % osmium tetroxide in 0.1 M phosphate buffer. The samples were embedded using the Spurr embedding kit, and sections were examined using a JEM-2100 electron microscope (JEOL, Tokyo, Japan).

### ALB analysis

Medium was harvested following 24 hours of culture for the different cell populations. The ALB content of the culture supernatants was quantified using a commercially available enzyme-linked immunosorbent assay (ELISA) kit (Alpha Diagnostic Intl, San Antonio, TX, USA) according to the manufacturer’s protocol.

### CYP activity assay

Ethoxyresorufin-*O*-deethylase (EROD), methoxyresorufin-*O*-deethylase (MROD), and pentoxyresorufin-*O*-deethylase (PROD) assays were performed to determine the activities of CYP1A1, CYP1A2, and CYP2B as described previously [21]. The differentiated cells were treated with 1 μM 7-ethoxyresorufin (Fanbo biochemical, Beijing, China), methoxyresorufin (Fanbo biochemical), and pentoxyresorufin (Sigma-Aldrich) for 24 hours, respectively. The fluorescent products of CYP450 substrates leaked from cells were determined at a wavelength of 585 nM in the absorption under Infnite® 200 PRO (Tecan, Männedorf, Switzerland). Quantitative studies employed the high-purity reference standard of resorufin (Fanbo biochemical) for assay standardization.

### Bile canaliculus analysis

The cells were incubated with 10 μM 5(6)-carboxy-2,7-dichlorofluorescein diacetate (CDFDA; Sigma-Aldrich) at 37 °C for 10 minutes to allow its internalization and subsequent translocation into the bile canaliculus (BC) lumen by MRP2 to determine the BC function. After extensive washes, the capacity of the BC to contain the fluorescent CDF was analyzed under a Leica microscope (Leica) as described previously [21].

### Measurement of the plasma amino transferase levels

The plasma alanine amino transferase (ALT) and aspartate aminotransferase (AST) levels were measured using a Mindray BS-200 analyzer (Mindray, Shenzhen, China).

### Acute liver failure induction in mice and hepatic tissue transplantation

Athymic nude BALB/c male mice aged 6–8 weeks received care according to the Capital Medical University guidelines. All protocols were approved by the Committee for Animal Care. The mice were injected with a single intraperitoneal dose of CCl4 solution in olive oil (5.0 ml/kg body weight as 1 %, vol/vol; Sigma-Aldrich) to induce acute liver injury [4]. Vehicle (olive oil)-injected mice (*n* = 3) were used as controls.

At 24 hours after CCl4 treatment, hASC-HLCs that were cultured on 3D-AHAM for 3 days and that contained 1 × 10^5^ cells were implanted at the edge of the superior right lobe of the liver with a 0.5 cm long incision under isoflurane anesthesia. 3D-AHAM pieces without cells were used as controls. The mice were sacrificed at days 1, 3, 7, 14, and 56 post implantation. In each group, there are three to six mice for transplantation. The CCl4 treatment was readministered at the end of another week for the 2-week group, and at the end of every week for the 8-week group. Serum ALT and AST levels were determined at the end of the procedure. Histological analysis of liver tissues was conducted by serial tissue sectioning and staining with hematoxylin & eosin (H&E), and the area of injury was assessed using ImageJ software (National Institutes of Health, Bethesda, MD, USA). Human nuclei, ALB, and HNF4α, CYP2B6, and mouse-specific CD31 expression were examined at different points by immunofluorescence.

### Drug metabolism activity assay

The drug metabolism activity was analyzed as described previously [23]. Ketoprofen (15 mg/kg; Sigma-Aldrich) was administrated intravenously to the mice post transplantation of the hASC-HLC–3D-AHAM graft or 3D-AHAM in the injured liver. Urine was collected 2 hours after administration. Then 100 μl urine was mixed with 100 μl of 0.5 M acetate buffer (pH 5.0), and 10 μl of 1 N KOH was added to urine samples, incubated at 80 °C for 3 hours, neutralized by 10 μl of 1 N HCl, and then centrifuged (15,000 rpm, 4 °C, 5 minutes). The supernatant was subjected to mass spectrometry (Quattro micro API; Waters, Milford, MA, USA). The ionspray voltage was –4500 V and the analyzed *m/z* transition (Q1/Q3) for ketoprofen, 1-hydroxyketoprofen, and glucuronide-conjugated ketoprofen was 253.06, 269.35, and 429.34, respectively.

### Statistical analysis

At least three independent determinations of each parameter were compared among the treatment groups by one-way analysis of variance using the statistical software SPSS 11.5 (IBM Corporation, Armonk, NY, USA). Differences were considered significant if *p* <0.05. All data are presented as the mean ± SD.

## Results

### AHAM retained the major components of the AM matrix

Fresh and treated HAM pieces were examined to establish whether the treatment successfully removed cellular components and to determine the decellularization process. The morphology of the AM surface under phase-contrast microscopy showed that no cells were visible in the treated (Figure S1B in Additional file 2) and cryopreserved (Figure S1C in Additional file 2) HAM pieces compared with the fresh HAM pieces (Figure S1A in Additional file 2). H&E staining confirmed that the decellularization process was successful (Figure S1E, F in Additional file 2), compared with the fresh HAM pieces (Figure S1D in Additional file 2). SEM analysis demonstrated that the histoarchitecture of the basement membrane was maintained and that no obvious disruption was present following decellularization and cryopreservation in AHAM (Figure S1H, I in Additional file 2), while a single layer of amnion epithelial cells were visible in the fresh HAM (Figure S1G in Additional file 2). Transmission electron microscopy (TEM) analysis demonstrated that a meshwork of collagenous fibrils and stroma were also preserved in AHAM (Figure S1J in Additional file 2).

The HAM pieces were then examined for the presence of major components of the ECM, including collagen type I, collagen type IV, fibronectin, and laminin, before and after decellularization and cryopreservation to determine whether the basement membrane proteins were retained following decellularization. Immunohistochemical analysis showed that these four types of components were all labeled by monoclonal antibodies (Additional file 3). Collagen type I and fibronectin staining were observed in the basement membrane and in the compact layer of the AHAM, and the distribution of collagen type IV and laminin was primarily in the surface of the basement membrane and appeared to be intact in a linear pattern. Therefore, we confirmed that the AHAM retained the natural architecture and components of the AM matrix after decellularization with trypsin–EDTA and cryopreservation with glycerol.

### AHAM promotes the functional maturation of the hASC-HLCs

**Fig. 1:**
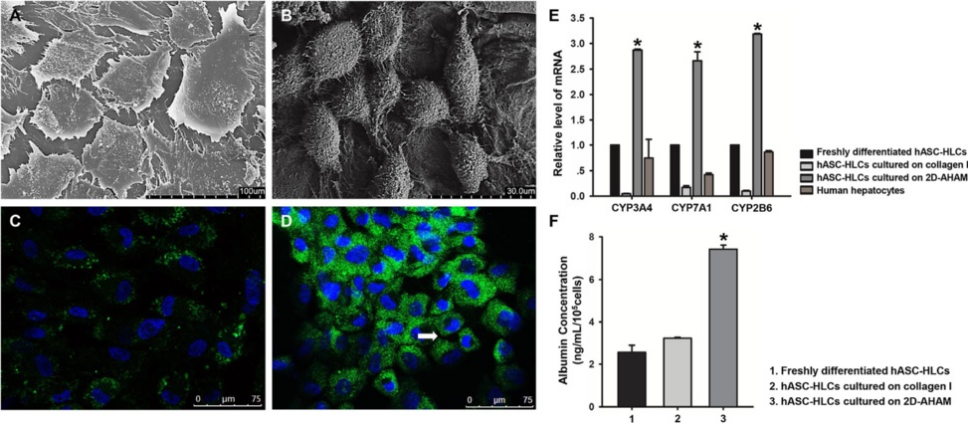
Properties of hASC-HLCs cultured on collagen type I-coated glass slides and on 2D-AHAM*.* SEM shows the morphology of hASC-HLCs cultured on collagen type I-coated glass slides (**a**) and on 2D-AHAM (**b**) for 72 hours *in vitro*. Immunofluorescence staining of human MRP2 of hASC-HLCs cultured on collagen type I-coated glass slides (**c**) and on 2D-AHAM (**d**). *Arrow* shows the location of the BC. **e** Real-time RT-PCR was used to analyze the expression of hepatocyte function-specific genes of hASC-HLCs plated on different substrates. The freshly differentiated hASC-HLCs (indicate that differentiated cells do not trypsinize and reseed after the end of the differentiated program of 21 days) and human hepatocytes were used as controls. The relative expression of each gene was normalized to 18S rRNA. *Statistically significant compared with the hASC-HLCs cultured on collagen type I (*p* <0.05). **f** Levels of ALB secreted by the hASC-HLCs cultured on different substrates as analyzed by ELISA. *CYP* cytochrome, *2D-AHAM* cryopreserved and dried acellular human amniotic membrane, *hASC* human adipose stem cell, *HLC* hepatocyte-like cell

The hASC-HLCs were seeded on collagen type I-coated cell culture plates and on 2D-AHAM. The morphology of the hepatocytes was then observed using phase-contrast microscopy at different time points to assess the biocompatibility of the AHAM. Within 2 hours after seeding, most of the cells cultured on collagen type I had adhered to the substrate and exhibited irregular shapes; however, the cells cultured on 2D-AHAM remained round. The cells cultured on 2D-AHAM began to adhere at approximately 6 hours after seeding and completely adhered to the AM matrix by 12 hours after seeding. By 72 hours of culture, the cells on collagen type I exhibited typical hepatocyte morphology with a polygonal shape; however, the cells on 2D-AHAM aggregated into clusters containing between 2 and 10 round cells (Additional file 4). Using SEM, the cells cultured on collagen type I appeared markedly flattened, with sharp edges and stiff protrusions (Fig. 1a); however, the morphology of the cells cultured on 2D-AHAM was clearly changed, with a smaller size, spheroidal shape, and abundant villi on the cell surface (Fig. 1b).

Immunofluorescence staining data verified that the cells on 2D-AHAM had significant staining for MRP2 (Fig. 1d), an apical membrane marker of hepatocytes, compared with the cells on collagen type I-coated plates (Fig. 1c). To evaluate the functional activity of drug transporters, the cells were cultured with CDFDA, a compound which is metabolized into a fluorescent marker, and transported by polarized cells via MRP2 into BC. The results showed that hASC-HLCs also formed a functional BC structure on the AHAM (Additional file 5).

Real-time RT-PCR analyses showed that the mRNA levels of hepatic metabolism functional markers, including CYP3A4, CYP7A1, and CYP2B6, in the cells cultured on 2D-AHAM were significantly higher than the freshly differentiated cells (in situ), cells on collagen type I, and primary human hepatocytes (Fig. 1e).

The level of ALB secreted by hASC-HLCs cultured on 2D-AHAM was also significantly higher than that produced by the freshly differentiated cells and the cells on collagen type I at 72 hours of culture (Fig. 1f). These results were consistent with the morphological observations, indicating that the AHAM has good biocompatibility with hepatocytes and promotes the functional maturation of hASC-HLCs.

### hASC-HLCs reorganized into a hepatic tissue-like structure on 3D-AHAM

**Fig. 2:**
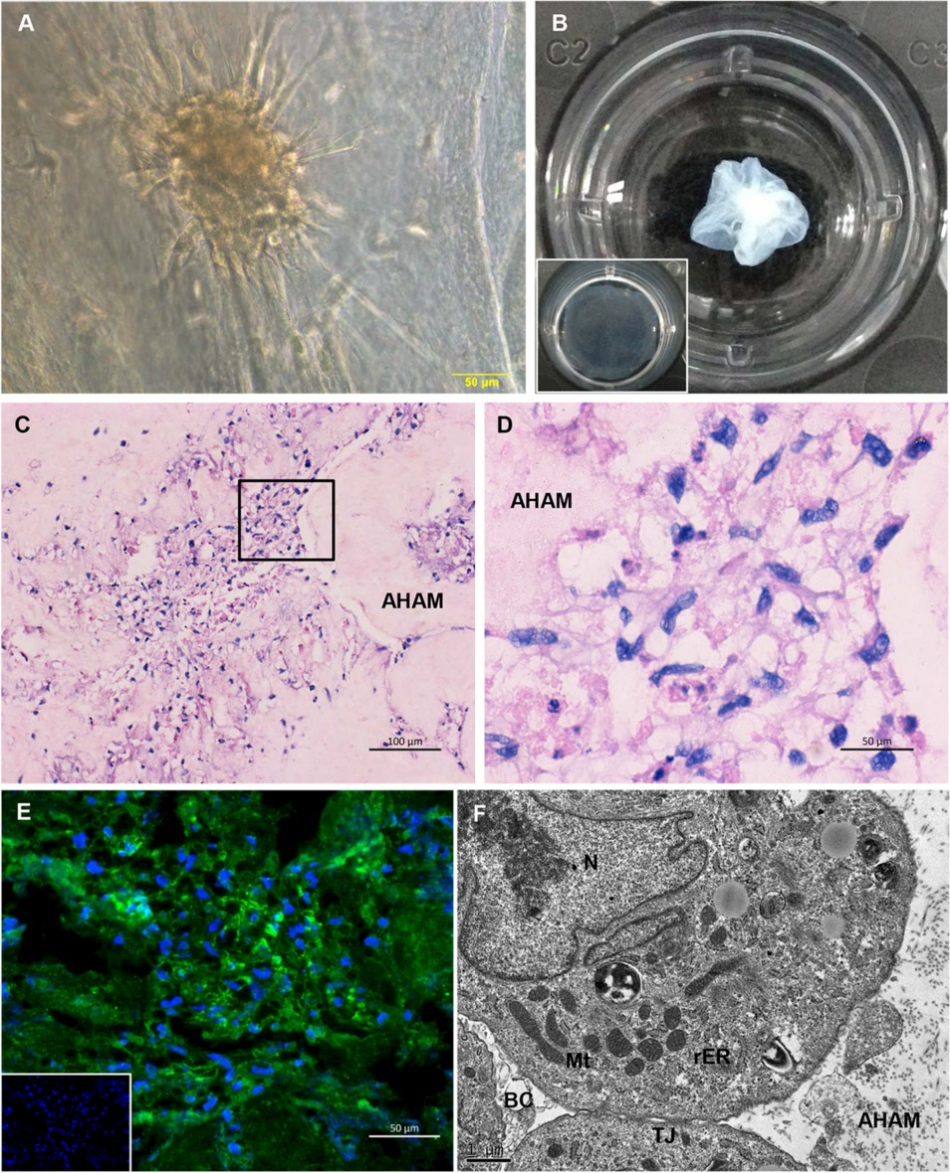
Properties of hASC-HLCs cultured on 3D-AHAM *in vitro* *.* Macroscopic appearance of the hASC-HLC–3D-AHAM cultured on day 1 (**a**) and day 3 (**b**) *in vitro*. Macroscopic appearance of the hASC-HLC–2D-AHAM cultured on day 3 *in vitro* (**b**) (*insert*). H&E staining showed that the hASC-HLCs attached to the AHAM intensely and formed a 3D nest-like structure (**c**, **d**) on day 3. Scale bars: 100 μm (**c**), 50 μm (**d**). **e** Immunofluorescence staining of ALB of hASC-HLC–3D-AHAM on day 3. The negative control using isotype IgG for immunofluorescence (**e**) (*insert*). Scale bar: 50 μm. **f** TEM analysis of the ultrastructure of hASC-HLC–3D-AHAM on day 3. Scale bar: 1 μm. *AHAM* acellular human amniotic membrane, *BC* bile canaliculus, *Mt* mitochondria, *N* nucleus, *rER* rough endoplasmic reticulum, *TJ* tight junction

hASC-HLCs were seeded on 3D-AHAM to assess the behaviors and functions of cells on natural AHAM. The cells aggregated into clusters on the AHAM at 24 hours of culture (Fig. 2a), and the cells on the smooth AHAM formed a 3D tissue-like structure by 72 hours of culture (Fig. 2b). Although the cells on 2D-AHAM also aggregated into small clusters after 72 hours of culture (Figure S3F in Additional file 4), they seldom organized into a 3D structure and the AHAM seemed to remain smooth (Fig. 2b insert). Routine histology, immunohistochemical staining, and TEM were performed to examine this 3D tissue-like structure. Upon histological analysis, the hepatocytes appeared well integrated into the AHAM and formed a net-like structure in the center of the tissue (Fig. 2c, d). Additionally, the majority of the cells were ALB-positive (Fig. 2e). The negative control using isotype IgG for immunofluorescence is shown in Fig. 2e (insert). TEM analysis confirmed that the hepatocytes in cultured hepatic tissues contained abundant mitochondria and that tight junctions and BC architecture formed between the adjacent hepatocytes (Fig. 2f).

**Fig. 3:**
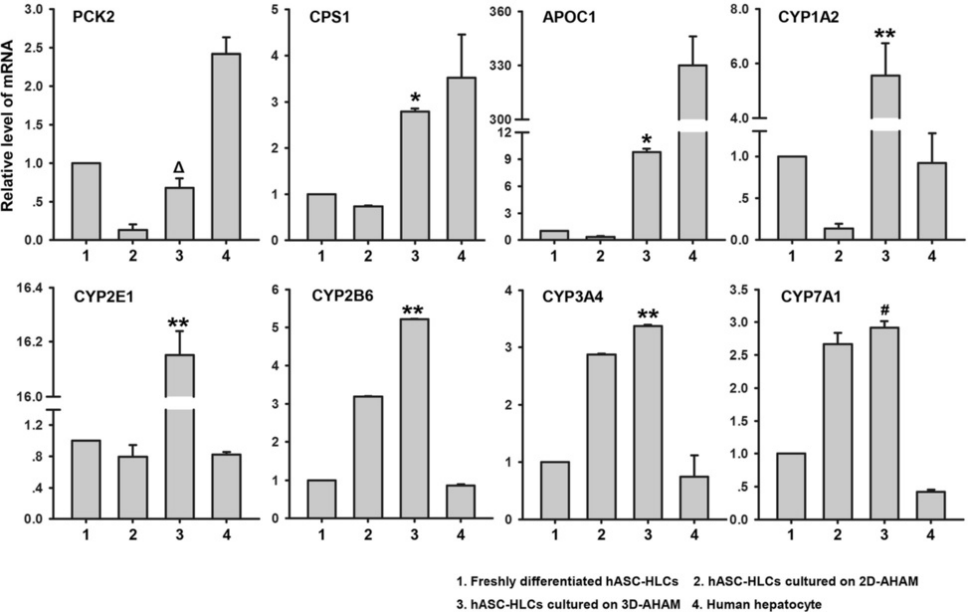
Expression levels of hepatocyte function-specific genes in hASC-HLCs plated on 2D-AHAM and 3D-AHAM. Real-time RT-PCR was used to analyze the expression of hepatocyte function-specific genes in hASC-HLCs plated on 2D-AHAM and 3D-AHAM (represented as 2D and 3D, respectively) on day 3. The freshly differentiated hASC-HLCs and human hepatocytes were used as controls. The relative expression of each gene was normalized to 18S rRNA. ^∆^Statistically significant compared with the 2D-AHAM (*p* <0.05). *Statistically significant compared with the 2D-AHAM and the freshly differentiated hASC-HLCs (*p* <0.05). **Statistically significant compared with the 2D-AHAM, the freshly differentiated hASC-HLCs, and human hepatocytes (*p* <0.05). ^#^Statistically significant compared with the freshly differentiated hASC-HLCs and human hepatocytes (*p* <0.05). *AHAM* acellular human amniotic membrane, *APOC1* apolipoprotein C-I, *CPS1* carbamoyl-phosphate synthase 1, *CYP1A2* cytochrome P450 1A2, *CYP2B6* cytochrome P450 2B6, *CYP2E1* cytochrome P450 2E1, *CYP3A4* cytochrome P450 3A4, *CYP7A1* cytochrome P450 7A1, *hASC* human adipose stem cell, *HLC* hepatocyte-like cell, *PCK2* phosphoenolpyruvate carboxykinase 2

Various hepatocyte characteristics were determined to investigate whether a 3D hepatic tissue-like culture was able to maintain the function of hepatocytes. The expression level of *PCK2* gene in the cells cultured on 3D-AHAM was higher than those of the cells cultured on 2D-AHAM, but lower than the freshly differentiated cells and primary human hepatocytes. The gene expression levels of *CPS1* and *APOC1* in the cells cultured on 3D-AHAM were significantly higher than those of the cells cultured on 2D-AHAM and the freshly differentiated cells, but lower than primary human hepatocytes. Interestingly, the gene expression levels of *CYP1A2*, *CYP2E1*, *CYP2B6*, and *CYP3A4* in the cells cultured on 3D-AHAM were significantly higher than those of the freshly differentiated cells, the cells cultured on 2D-AHAM, and primary human hepatocytes. The gene expression levels of *CYP7A1* in the cells cultured on 3D-AHAM were significantly higher than those of the freshly differentiated cells and primary human hepatocytes, but there was no difference between the cells cultured on 2D-AHAM and 3D AHAM (Fig. 3).

**Fig. 4:**
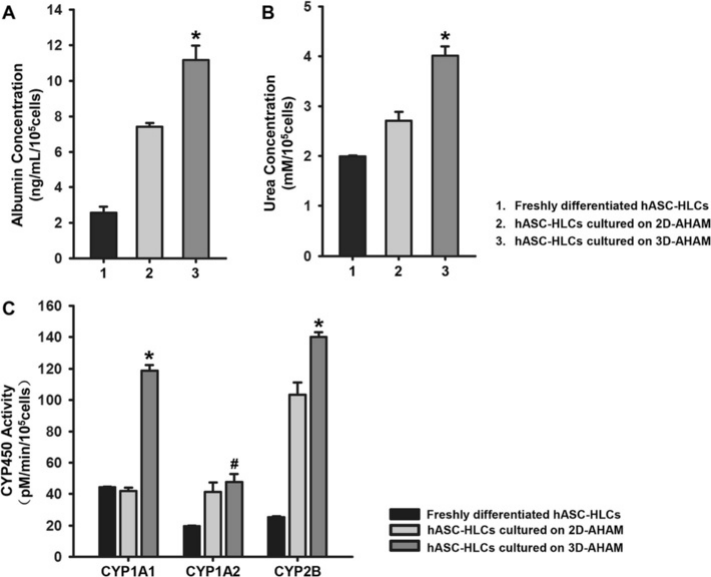
Functional properties of hASC-HLCs on 3D-AHAM *in vitro.* **a** ALB secretion of hASC-HLCs cultured on 2D-AHAM and 3D-AHAM, and freshly differentiated hASC-HLCs (represented as 1, 2, and 3, respectively) analyzed by ELISA. **b** Ammonia metabolism, as reflected by urea synthesis, was determined in the hASC-HLCs cultured on 2D-AHAM and 3D-AHAM, and freshly differentiated hASC-HLCs (represented as 1, 2, and 3, respectively). **c** EROD, MROD, and PROD assays for the activities of CYP1A1, CYP1A2, and CYP2B in hASC-HLCs cultured on 2D-AHAM and 3D-AHAM, and freshly differentiated hASC-HLCs. *Statistically significant compared with the hASC-HLCs cultured on 2D-AHAM and freshly differentiated hASC-HLCs (*p* <0.05). ^#^Statistically significant compared with the freshly differentiated hASC-HLCs (*p* <0.05). *AHAM* acellular human amniotic membrane, *CYP* cytochrome, *hASC* human adipose stem cell, *HLC* hepatocyte-like cell

ALB secretion, ammonia detoxification, and CYP450 activity were detected at day 3 of culture to evaluate the functional activity of hASC-HLCs on 2D-AHAM and 3D-AHAM. The ALB level (11 ng/10^5^ cells/day) secreted by hASC-HLCs cultured on 3D-AHAM was higher than the level secreted by the freshly differentiated cells, and the cells on 2D-AHAM at 72 hours of culture (Fig. 4a). Similarly, the level of urea production in hASC-HLCs cultured on 3D-AHAM was also higher than that of the freshly differentiated cells and the cells on 2D-AHAM (Fig. 4b). The results presented in Fig. 4c indicate that the activities of CYP1A1 and CYP2B in hASC-HLCs cultured on 3D-AHAM were significantly higher compared with those in hASC-HLCs cultured on 2D-AHAM and the freshly differentiated cells. The activities of CYP1A2 in hASC-HLCs cultured on 3D-AHAM were higher than the freshly differentiated cells, but there was no difference between the cells cultured on 3D-AHAM and 2D-AHAM (Fig. 4c).

Taken together, these results suggested that the function of hASC-HLCs cultured on 3D-AHAM was relatively higher than that of the hASC-HLCs cultured on the 2D-AHAM. Moreover, the 3D-AHAM culture condition is likely to be more similar to the *in vivo* condition than the monolayer culture condition.

### hASC-HLC–3D-AHAM constructs implant into the livers of immunodeficient mice

The hASC-HLC–3D-AHAM constructs were implanted into an immunodeficient mouse model with CCl4 treatment to test whether the hASC-HLC–3D-AHAM constructs can engraft after transplantation. The acute liver injury induced by a single-dose injection of CCl4 was evidenced by an increase in the plasma ALT level to >2500 U/l (normal range, <50 U/l), by an increase in the plasma AST level to >2200 U/l (normal range, <80 U/l), and by larger necrotic areas around the central venous as determined by histochemistry analysis 24 hours after CCl4 treatment (Additional file 6).

**Fig. 5:**
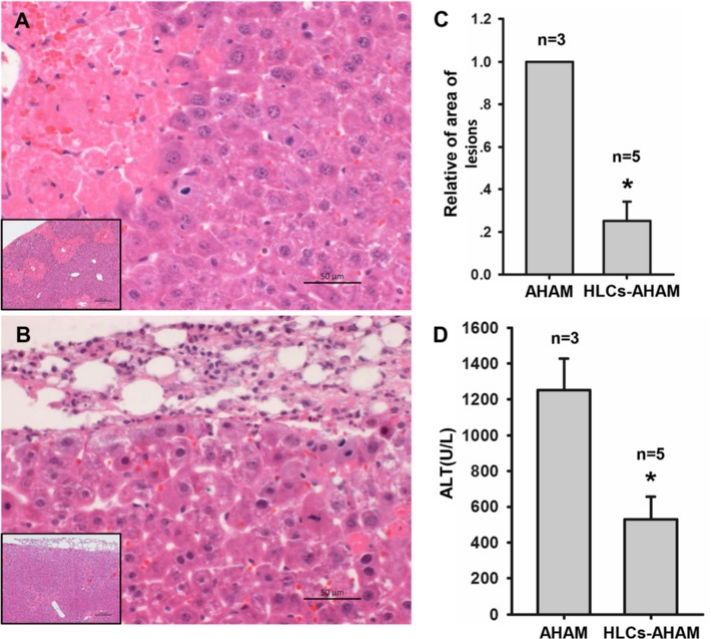
Semiquantitative evaluation of lesions in liver tissue sections following transplantation. H&E staining analysis of the lesions in the liver tissue at 24 hours after the transplantation of AHAM (**a**) (*n* = 3) or hASC-HLC–3D-AHAM grafts (**b**) (*n* = 5) in a CCl4-induced liver injury immunodeficient mouse model. **c** Percentage of lesion area was evaluated using Image J software. **d** Plasma levels of ALT were detected. *Statistically significant compared with the AHAM graft (*p* <0.05). *AHAM* acellular human amniotic membrane, *ALT* alanine amino transferase, *HLC* hepatocyte-like cell

Acute liver injury was validated by analyzing the massive necrosis during histological examination and the serum ALT levels in the injured mice with the hASC-HLC–3D-AHAM construct or with AHAM only to evaluate the beneficial effect of the hASC-HLC–3D-AHAM construct on the recovery of the CCl4-injured liver after implantation. H&E staining (Fig. 5a, b) showed that the areas of inflammation, sinusoid congestion, and hemorrhage decreased and that the serum ALT level decreased significantly in mice implanted with hASC-HLC–3D-AHAM constructs compared with the mice implanted with AHAM only (Fig. 5c, d). Interestingly, the transplanted AHAM only attached the middle of the liver lobes and did not integrate with the liver tissue (Fig. 5a). In contrast, approximately 33 % of the mice (*n* = 6) displayed integrated hASC-HLC–3D-AHAM transplants (Fig. 5b). The results suggested that the hASC-HLC–3D-AHAM engraftment might alleviate the liver injury induced by CCl4.

**Fig. 6:**
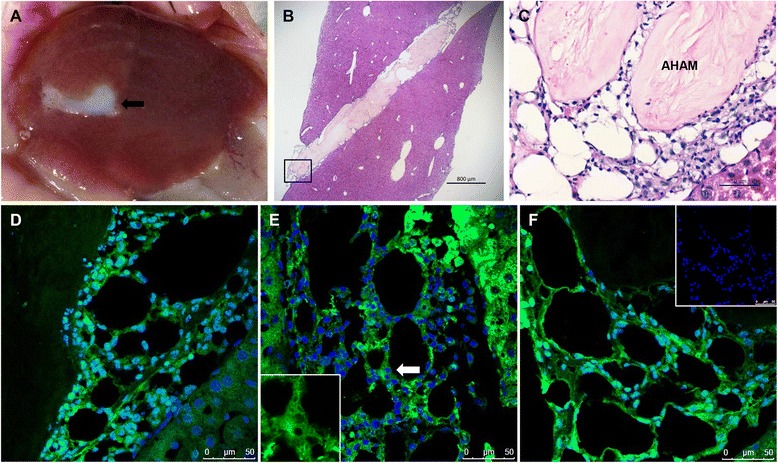
Implantation of the hASC-HLC–3D-AHAM graft into the CCl4-injured liver. **a** Gross appearance of the hASC-HLC–3D-AHAM graft in the injured liver at 3 days post implantation. *Arrow* shows the location of the graft. **b**, **c** H&E staining analysis of the graft integrated with the liver tissue. Scale bars: 800 μm **b**, 50 μm **c**. Immunofluorescence staining of human nuclei (**d**), ALB (**e**), and HNF4α (**f**) in the hASC-HLC–3D-AHAM graft in the CCl4-injured liver. Scale bar: 50 μm. Negative control using isotype IgG for immunofluorescence (**f**) (*insert*). *AHAM* acellular human amniotic membrane

Histological and immunohistochemical analyses were performed at days 3, 7, and 14 post transplantation to detect the engraftment of the transplanted hASC-HLC–3D-AHAM constructs. The results showed that the hASC-HLC–3D-AHAM constructs had engrafted into the livers of the mice at day 3 post transplantation (Fig. 6a). H&E staining showed that the tissue-like constructs were restricted to the cut edge of each liver and that the cells in this construct formed large net-like structures (Fig. 6b, c). Immunohistochemical analysis confirmed that the cells in the construct stained positive for human nuclei, ALB, and HNF4α (Fig. 6d–f). Data in Additional files 7 and 8 reveal that the hASC-HLC–3D-AHAM constructs remained integrated into the liver at days 7 and 14 post transplantation, with large clusters of hepatocytes arranged into cord-like structures, and the cells in the construct were also positive for human nuclei, ALB, and HNF4α. The results suggested that the AHAM engraftment of hASC-HLCs *in vivo* was highly efficient.

**Fig. 7:**
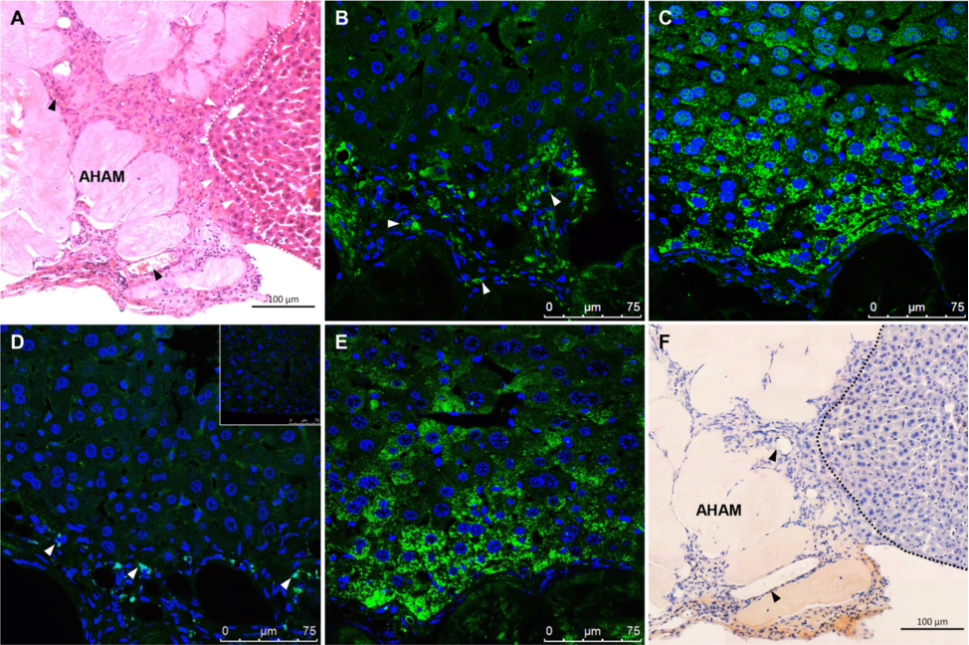
Long-term properties of the hASC-HLC–3D-AHAM graft in the injured liver following transplantation*.* **a** H&E staining analysis of the graft at 8 weeks post implantation. *Black arrows* show the location of the vessel-like structure. *White arrows* show the hASC-HLCs with the cubic morphology. Immunofluorescence staining of human nuclei (**b**), ALB (**c**), HNF4α (**d**), CYP2B6 (**e**), and mouse CD31 (**f**) in the hASC-HLC–3D-AHAM graft in the CCl4-injured liver at 8 weeks post implantation. The control of immunofluorescence using anti-human nuclei was analyzed in tissues from nontransplanted mice (**d**) (*insert*). *Black arrows* show the location of the vessel-like structure. *Dotted lines* show the location of the grafts. Scale bar: 75 μm (**b**), (**c**), 100 μm (**a**), (**d**). *AHAM* acellular human amniotic membrane

The fate of transplanted hASC-HLC–3D-AHAM constructs in mice was considered in the long term. H&E staining showed that the tissue-like constructs were completely integrated to the liver at day 56 post transplantation, and the morphology of cells in grafts near the edge of the host changed to cubic (Fig. 7a). There was no evidence of abnormal proliferation or regression in the graft. Immunohistochemical analysis confirmed that the cells in the construct stained positive for human nuclei (Fig. 7b), and for hepatic functional marker ALB (Fig. 7c), HNF4α (Fig. 7d), and CYP2B6 (Fig. 7e).

The functional maturation of hASC-HLCs in transplants was evaluated using the drug metabolism activity assay. Mice transplanted with the hASC-HLC–3D-AHAM constructs or 3D-AHAM were challenged with ketoprofen, which are known to be metabolized differently by mice and humans. After the drug exposure, formation of metabolites in urine samples from mouse or human specific was collected and determined. Results showed that the 1-hydroxyketoprofen was detected in mice transplanted with hASC-HLC–3D-AHAM constructs or 3D-AHAM. However, the glucuronide-conjugated ketoprofen was only detected in the urea sample of mice transplanted with hASC-HLCs at day 56 post transplantation (Additional file 9). Interestingly, H&E staining showed that the vessel-like structures presented in the transplanted hASC-HLC–3D-AHAM graft (Fig. 7a). Immunohistochemical analysis confirmed that cells composed the vessel-like structures were positive for mouse CD31 antibody (Fig. 7f). These results demonstrate that hASC-HLCs maintained the property of functional maturation, and the potential functional vessels in grafts were connecting with host vessels when hASC-HLC–3D-AHAM grafts were transplanted in the long term.

Our study has demonstrated that hASC-HLC–3D-AHAM transplantation offers an alternative approach to the generation of a 3D, transplantable hepatic graft. These results offered the therapeutic potential using autohepatocytes derived from patient stem cells for treating liver failure.

## Discussion

The use of hASC-derived hepatocytes as regenerative cells in hepatic disease would be advantageous for ethical and safety reasons, and these autologous cells are immunocompatible, exhibit controlled differentiation, and do not undergo post-transplantation rejection [3]. Thus far, the low engraftment of the available hepatocytes has been a major obstacle [24]. Recently, hepatocytes that have been assembled on 3D decellularized matrices to generate liver tissues or patches have become a novel platform for transplantation [14, 25, 26]. In the present study, we provide evidence that the AHAM has good biocompatibility with hepatocytes and promotes the functional maturation of hASC-HLCs. Moreover, the natural AHAM may induce the reorganization of hASC-HLCs into a hepatic tissue-like structure *in vitro*, which efficiently alleviated the acute liver injury, was integrated with the liver tissue at the implantation site, and induced hepatic plate-like structure in the grafts after transplantation into CCl4-injured livers of immunodeficient mice.

The AM, which is the innermost layer of the placenta, is composed of an amniotic epithelial cell layer, a basement membrane, and a thick compact layer or avascular stroma. The special structure and biological viability of the amniotic ECM plays a major role in providing mechanical strength to the amniotic epithelial cells [18]. Previous evidence showed that the acellular matrix nature of AM also functions as a primary determinant of attachment, survival, differentiation, and cytoskeletal organization for epithelial cells of other origins [17], such as pneumocytes [27], limbal epithelial cells [28, 29], salivary gland cells [30], and dental apical papilla cells [31]. The AHAM may therefore be a reasonable native scaffold for hepatocyte seeding.

Retaining the biochemical composition and architecture of the matrix in intact AM following the decellularization process is a critical step for natural scaffold preparation. First, we demonstrated that the amniotic epithelial cells were removed completely from the AM using 0.25 % trypsin and 0.38 % EDTA without any mechanical scraping [16]. The architecture of the AM matrix, including a smooth and compact collagenous basement surface and a meshwork of collagenous fibrils and stroma, was preserved upon decellularization and cryopreservation (Additional file 2). Immunostaining analysis suggested that the matrix of collagen type I, collagen type IV, fibronectin, and laminin found in the hepatocyte niche remained relatively intact compared with that of the fresh AM (Additional file 3). Then, we compared the morphological appearance and function of hASC-HLCs when seeded on conventional collagen type I-coated plates and 2D-AHAM configurations. The results showed that the hASC-HLCs cultured on 2D-AHAM appeared round or spheroidal, which is the desirable morphology for the functional maintenance of hepatocytes, versus the flattened and spread polygonal cells cultured on collagen type I as shown previously (Fig. 1) [21]. Consistent with these findings, quantitative gene expression analysis illustrated that hASC-HLCs cultured on 2D-AHAM exhibited a significant increase in hepatocyte function-specific gene expression at day 3 than the freshly differentiated cells, the cells cultured on collagen type I, and human hepatocytes (Fig. 1e). An analogous increase in functional properties, including CDFDA metabolism and ALB secretion, was observed in hASC-HLCs cultured on 2D-AHAM (Fig. 1; Additional file 5). These data demonstrated that the matrix components of AHAM have an effect on maintaining the hepatocyte phenotype. The accumulated evidence suggested that both collagen types I and IV, as well as the widely distributed adhesive protein fibronectin, play an important role in modulating the formation of hepatocyte polarity, such as the organization of cytoskeletal proteins and the expression of a wide array of liver-specific functions [32]. The AHAM may also be a favorable substrate for hepatocyte carriers.

Next, we examined whether hASC-HLCs could attach to the natural AM ECM (3D-AHAM), which not only preserved the components of the AM matrix but also retained the specific 3D architecture of the AM matrix. The results showed that hASC-HLCs self-organized into microscopically visible 3D cell clusters by an intrinsic organizing capacity after 24 hours of culture on 3D-AHAM. The macroscopically visible clusters continued to form for up to 72 hours of culture (Fig. 2). We visualized the formation of homogeneously distributed hASC-HLCs in this construct as detected by histology, immunostaining, and TEM. Similarly, the levels of ALB secretion, urea synthesis, and CYP450 enzyme activity were elevated in hASC-HLCs grown on 3D-AHAM compared with their counterparts grown on 2D-AHAM and the freshly differentiated cells (Fig. 4). This result suggests that the 3D-AHAM microenvironment or spatial organization may provide more effective signals, such as the surface texture, variation in pore diameter, spatial presentation of the surface proteins, and mechanical strength for the hASC-HLCs [32, 33].

The efficiency of the hASC-HLC–3D-AHAM constructs for hepatocyte transplantation was further assessed using a grafting protocol. Our study dramatically demonstrated that the hASC-HLC–3D-AHAM engraftment alleviated the acute liver injury on day 1 and the efficiency was restricted to the desired target sites on day 3 post transplantation as determined by histological examination. The fate of transplanted hASC-HLC–3D-AHAM constructs was further examined 2 months post transplantation. Similarly, the constructs were integrated in the liver of mice as before without multiplying and regressively repopulating. For assessing progressive humanization or the functional maturation of hASC-HLCs in host liver, the ketoprofen metabolism was performed *in vivo* to validate the functionality of engrafted cells and the functional vessel formation in transplanted hASC-HLC–3D-AHAM constructs. Ketoprofen is a propionic acid-class nonsteroidal anti-inflammatory drug with analgesic and antipyretic effects, which are known to be metabolized differently by mice and humans [23, 34]. Ketoprofen is primarily metabolized by CYP450s in mice to form 1-hydroxyketoprofen, while in humans ketoprofen is mainly metabolized by UDP-glucuronosyltransferase to form ketoprofen glucuronide by hydrolysis [35]. We observed that the glucuronide-conjugated ketoprofen was detected in urea in mice transplanted with hASC-HLC–3D-AHAM using mass spectrometry. The histological and immunohistochemical analysis further demonstrated the functional vessel structure formed in the transplanted hASC-HLC–3D-AHAM constructs.

## Conclusions

The present study suggests that the AHAM has the ability to maintain the functional phenotype of the hASC-HLCs and is transplantable. This approach sounds rather straightforward; alternatives that would aid in providing surgical potential to manage the bottleneck of donor liver availability for both organ and hepatocyte transplantation are therefore worth discussing. Our findings, combined with the development of tissue engineering technologies, may support adult stem cell-based therapy for future individualized clinical therapy of liver failure and for bioartificial liver establishment.

## Additional files

Additional file 1:
**is Table S1 presenting primers for real-time RT-PCR.** (DOC 35 kb) Additional file 2:
**is Figure S1 showing characterization of fresh HAM and AHAM.** The properties of fresh HAM (A, D, G), fresh AHAM (B, E, H), and cryopreserved AHAM (C, F, I) were determined by phase microscopy (A, B, C), H&E staining (D, E, F), SEM (G, H, I) and TEM (J). Scale bars: 50 μm A–F. (TIFF 7208 kb) Additional file 3:
**is Figure S2 showing distribution of ECM components in fresh HAM and in cryopreserved AHAM.** Immunohistological evaluation of fresh HAM and cryopreserved AHAM before application. Scale bars: 50 μm. *HAM* human amniotic membrane, *AHAM* acellular human amniotic membrane, *I* collagen type I, *IV* collagen type IV, *FN* fibronectin, *LN* laminin. (TIFF 9626 kb) Additional file 4:
**is Figure S3 showing morphological properties of hASC-HLCs on different matrices.** Morphology of hASC-HLCs cultured on collagen type I-coated 24-well cell culture plates (A, C, E) and on cryopreserved 2D-AHAM (B, D, F) was examined by phase microscopy at 6 hours (A, B), 12 hours (C, D) and 72 hours (E, F) after cell seeding. Scale bars: 50 μm. (TIFF 9815 kb) Additional file 5:
**is Figure S4 showing BC analysis.** CDFDA is internalized by hASC-HLCs cultured on collagen type I-coated glass slides (A) and on 2D-AHAM (B), cleaved by intracellular esterases, and excreted into the BC as fluorescent CDF. *Arrow* shows the location of the fluorescent CDF. Scale bars: 50 μm. (TIFF 5628 kb) Additional file 6:
**is Figure S5 showing acute liver injury in BALB/c nude mice induced with CCL4.** The general appearance of the liver (A) and H&E staining (B) in BALB/c nude mice that were intraperitoneally injected with olive oil. Gross appearance of the liver (C) and H&E staining (D) in BALB/c nude mice that were intraperitoneally injected with CCL4. At 24 hours post injection, white spots were found at the surface of the liver (C), and H&E staining (D) showed more numerous and larger necrotic areas in the liver around the central venous (*black arrow*). Scale bar: 100 μm. Plasma levels of ALT (E) and AST (F) were detected in the CCl4-induced acute liver injury group and in the control. *Statistically significant compared to the control group (*p* <0.05). (TIFF 7839 kb) Additional file 7:
**is Figure S6 showing properties of the hASC-HLC–3D-AHAM graft in the injured liver at day 7 following transplantation.** (A) H&E staining analysis of the graft at day 7 post implantation. Scale bar: 100 μm. Immunofluorescence staining of human nuclei (B), ALB (C), and HNF4α (D) in the hASC-HLC–3D-AHAM graft in the CCL4-injured liver at 1 week post implantation. IgG as negative control (B, *insert*). Scale bar: 50 μm. (TIFF 9840 kb) Additional file 8:
**is Figure S7 showing properties of the hASC-HLC–3D-AHAM graft in the injured liver at day 14 following transplantation.** (A) H&E staining analysis of the graft at day 14 post implantation. Immunofluorescence staining of human nuclei (B), ALB (C), and HNF4α (D) in the hASC-HLC–3D-AHAM graft in the CCL4-injured liver at 2 weeks post implantation. IgG as negative control (B, *insert*). Scale bar: 50 μm. (TIFF 9846 kb) Additional file 9:
**is Figure S8 showing drug metabolism activity of the hASC-HLC–3D-AHAM graft in the mouse liver at day 56 post implantation.** Ketoprofen (15 mg/kg; Sigma-Aldrich) was administrated intravenously to the mice post transplantation of the hASC-HLC–3D-AHAM graft (A, B, C) or 3D-AHAM (D, E, F) in the injured liver. Urine was collected 2 hours after administration. Then 100 μl urine was mixed with 100 μl of 0.5 M acetate buffer (pH 5.0), and then 10 μl of 1 N KOH was added to urine samples, incubated at 80 °C for 3 hours, neutralized by 10 μl of 1 N HCl, and then centrifuged (15,000 rpm, 4 °C, 5 minutes). The supernatant was subjected to mass spectrometry (Quattro micro API; Waters). The ionspray voltage was –4500 V and analyzed *m/z* transition (Q1/Q3) for ketoprofen,1-hydroxyketoprofen, glucuronide-conjugated ketoprofen was 253.06, 269.35, and 429.34, respectively. (B) Amplification of part 1, (C) amplification of part 2, (E) amplification of part 3, (F) amplification of part 4. *Arrow* shows the location of the ketoprofen, 1-hydroxyketoprofen, glucuronide-conjugated ketoprofen, OH-ketoprofen: 1-hydroxyketoprofen; G-ketoprofen: glucuronide-conjugated ketoprofen. (TIFF 9829 kb)

## Abbreviations

AHAM: Acellular human amniotic membrane
ALB: Albumin
ALT: Alanine amino transferase
AM: Amniotic membrane
APOC1: Apolipoprotein C-I
AST: Aspartate aminotransferase
BC: Bile canaliculus
CCl4: Carbon tetrachloride
CDFDA: 5(6)-Carboxy-2,7-dichlorofluorescein diacetate
CPS1: Arbamoyl-phosphate synthase 1
CYP: Cytochrome
2D: Two-dimensional
3D: Three-dimensional
DAPI: 4′,6-Diamidino-2-phenylindole
ECM: Extracellular matrix
EDTA: Ethylenediamine tetraacetic acid
ELISA: Enzyme-linked immunosorbent assay
EROD: Ethoxyresorufin-*O*-deethylase
HAM: Human amniotic membrane
hASC: Human adipose stem cell
H&E: Hematoxylin & eosin
HLC: Hepatocyte-like cell
HNF: Hepatocyte nuclear factor
MROD: Methoxyresorufin-*O*-deethylase
MRP2: Multidrug resistance-related protein 2
PBS: Phosphate-buffered saline
PCK2: Phosphoenolpyruvate carboxykinase 2
PROD: Pentoxyresorufin-*O*-deethylase
SD: Standard deviation
SEM: Scanning electron microscopy
TEM: Transmission electron microscopy
