# Supplementary material for: Transplantation of human adipose stem cell-derived hepatocyte-like cells with restricted localization to liver using acellular amniotic membrane
Source: Stem Cell Res Ther. 2015 Nov 5;6:217. doi: 10.1186/s13287-015-0208-9 (PMC4635993; doi:10.1186/s13287-015-0208-9)
Supplement: Additional file 1: — is Table S1 presenting primers for real-time RT-PCR. (DOC 35 kb) [file 13287_2015_208_MOESM1_ESM.doc]

Table S1. Primers for Real-Time RT-PCR

| Accession number | Name | 5’-Sequence-3’ |
| --- | --- | --- |
| NM_001018073.1 | PCK2 | F: TTC CCC ACC GCA CAT ACC  R: CCA CCA CCA ATC CCA ACG |
| NM_001875.4 | CPS1 | F: CTG ACC CTG CCT ACA AAG  R: CAC CAG CAA ACC TGA AAC |
| NM_001645.3 | APOC1 | F: GGT CCT GGT GGT GGT TCT  R: TGT TTG ATG CGG CTG ATG |
| NM_000761.3 | CYP1A2 | F: AGT CTG TTC CCT TCT CGG  R: GGC TCT GGT GGA CTT TT |
| NM_001202855.2 | CYP3A4 | F: GGC GGA TGT TGA AGT GAG  R: GTT GGG TGT TGA GGA TGG |
| NM_000780 | CYP7A1 | F: GAG AAG GCA AAC GGG TGA AC  R: GCA CAA CAC CTT ATG GTA TGA CA |
| NM_000767 | CYP2B6 | F: TCT TCC AGT CCA TTA CCG CCA ACA  R: GCC GAA TAC AGA GCT GAT GAG TGA |
| NM_000773 | CYP2E1 | F: CTG ACC ACC CTC CGG AAC TAT  R: GGC CTT GGG TCT TCC TGA GT |
|  | 18S | F: GTA ACC CGT TGA ACC CCA TT  R: CCA TCC AAT CGG TAG TAG CG |
