# Supplementary figures and images for: Transplantation of human adipose stem cell-derived hepatocyte-like cells with restricted localization to liver using acellular amniotic membrane
Source: Stem Cell Res Ther. 2015 Nov 5;6:217. doi: 10.1186/s13287-015-0208-9 (PMC4635993; doi:10.1186/s13287-015-0208-9)

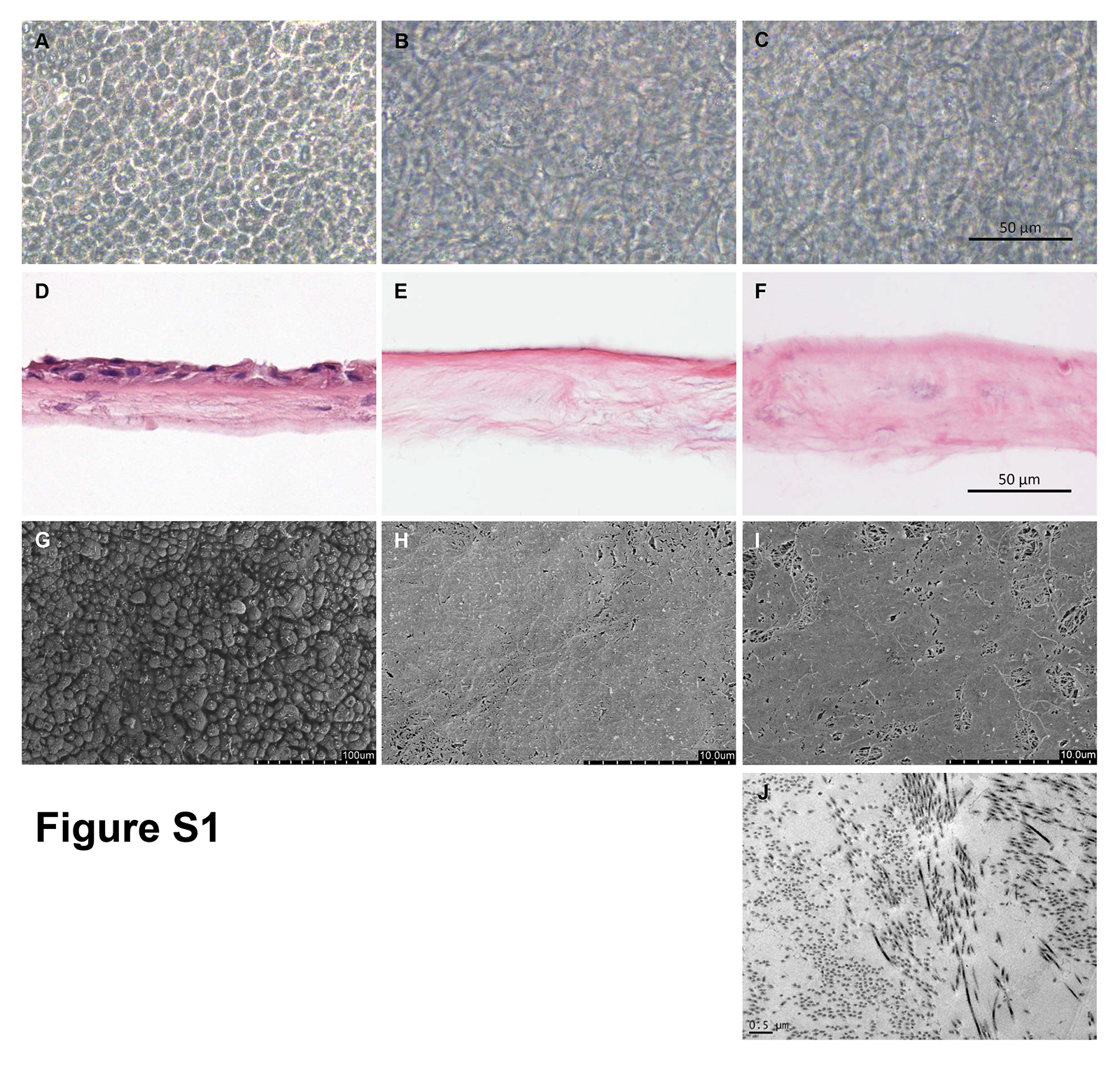

Supplement: Additional file 2: — is Figure S1 showing characterization of fresh HAM and AHAM. The properties of fresh HAM (A, D, G), fresh AHAM (B, E, H), and cryopreserved AHAM (C, F, I) were determined by phase microscopy (A, B, C), H&E staining (D, E, F), SEM (G, H, I) and TEM (J). Scale bars: 50 μm A–F. (TIFF 7208 kb) [file 13287_2015_208_MOESM2_ESM.tiff]

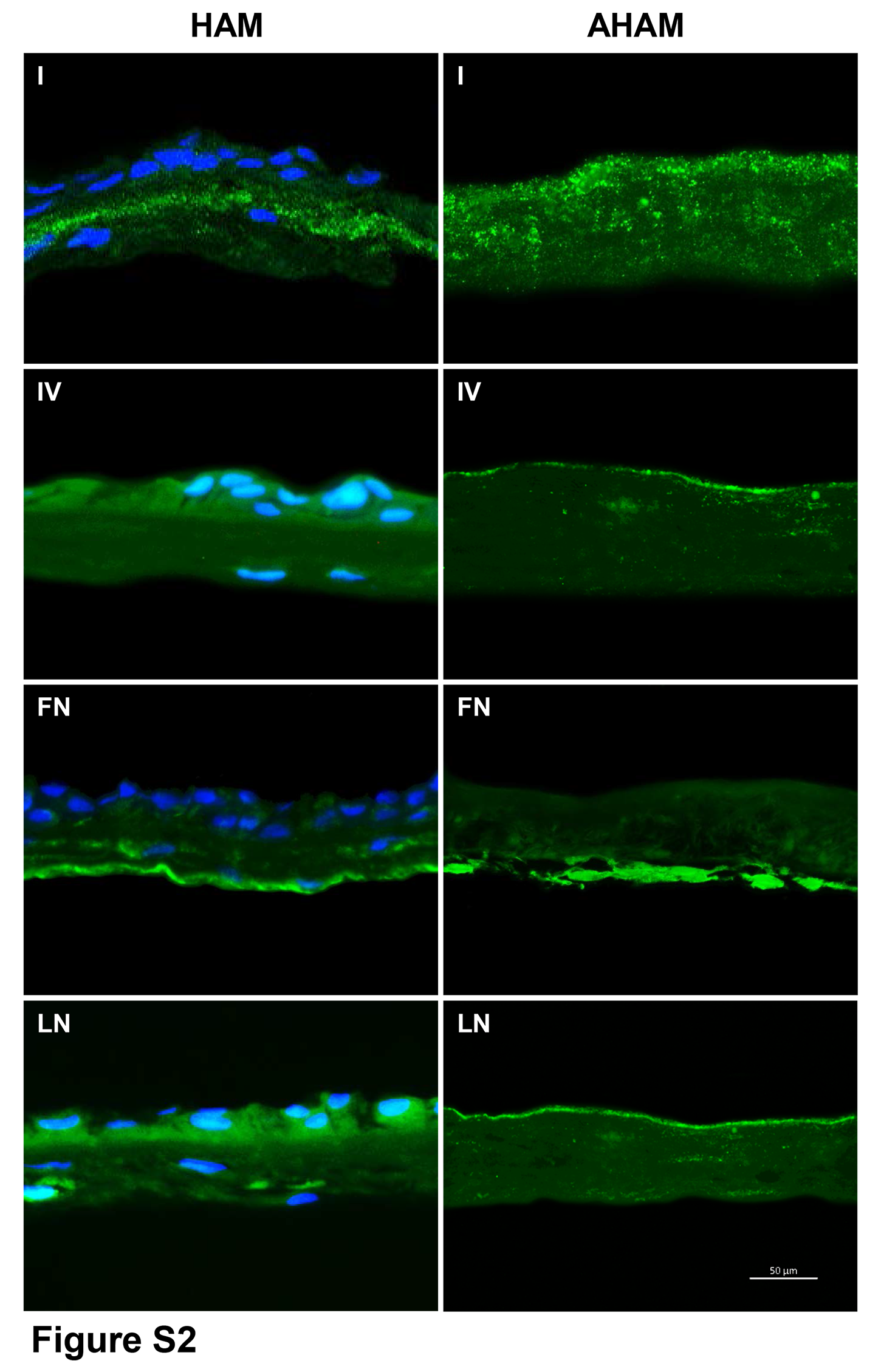

Supplement: Additional file 3: — is Figure S2 showing distribution of ECM components in fresh HAM and in cryopreserved AHAM. Immunohistological evaluation of fresh HAM and cryopreserved AHAM before application. Scale bars: 50 μm. HAM human amniotic membrane, AHAM acellular human amniotic membrane, I collagen type I, IV collagen type IV, FN fibronectin, LN laminin. (TIFF 9626 kb) [file 13287_2015_208_MOESM3_ESM.tiff]

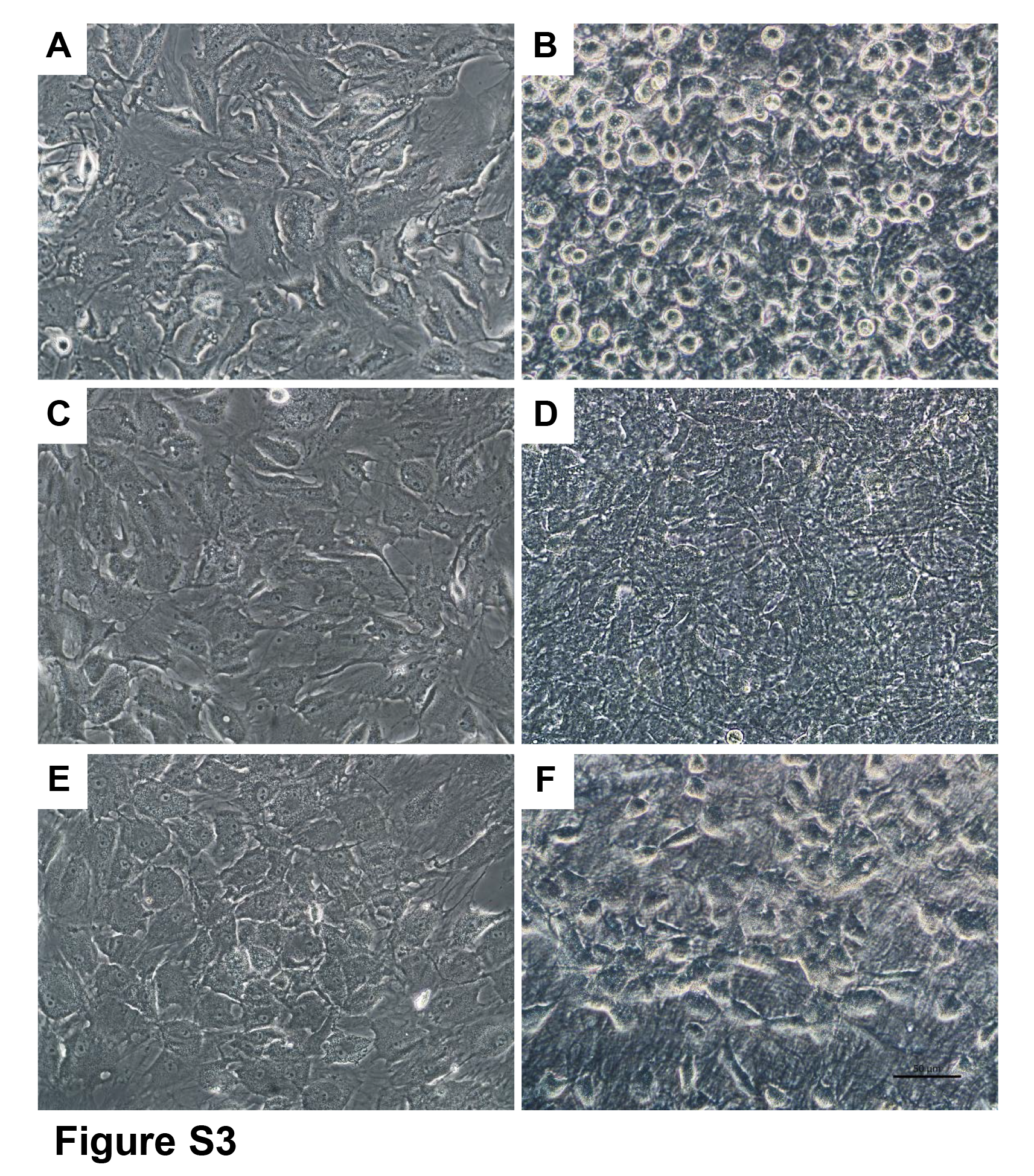

Supplement: Additional file 4: — is Figure S3 showing morphological properties of hASC-HLCs on different matrices. Morphology of hASC-HLCs cultured on collagen type I-coated 24-well cell culture plates (A, C, E) and on cryopreserved 2D-AHAM (B, D, F) was examined by phase microscopy at 6 hours (A, B), 12 hours (C, D) and 72 hours (E, F) after cell seeding. Scale bars: 50 μm. (TIFF 9815 kb) [file 13287_2015_208_MOESM4_ESM.tiff]

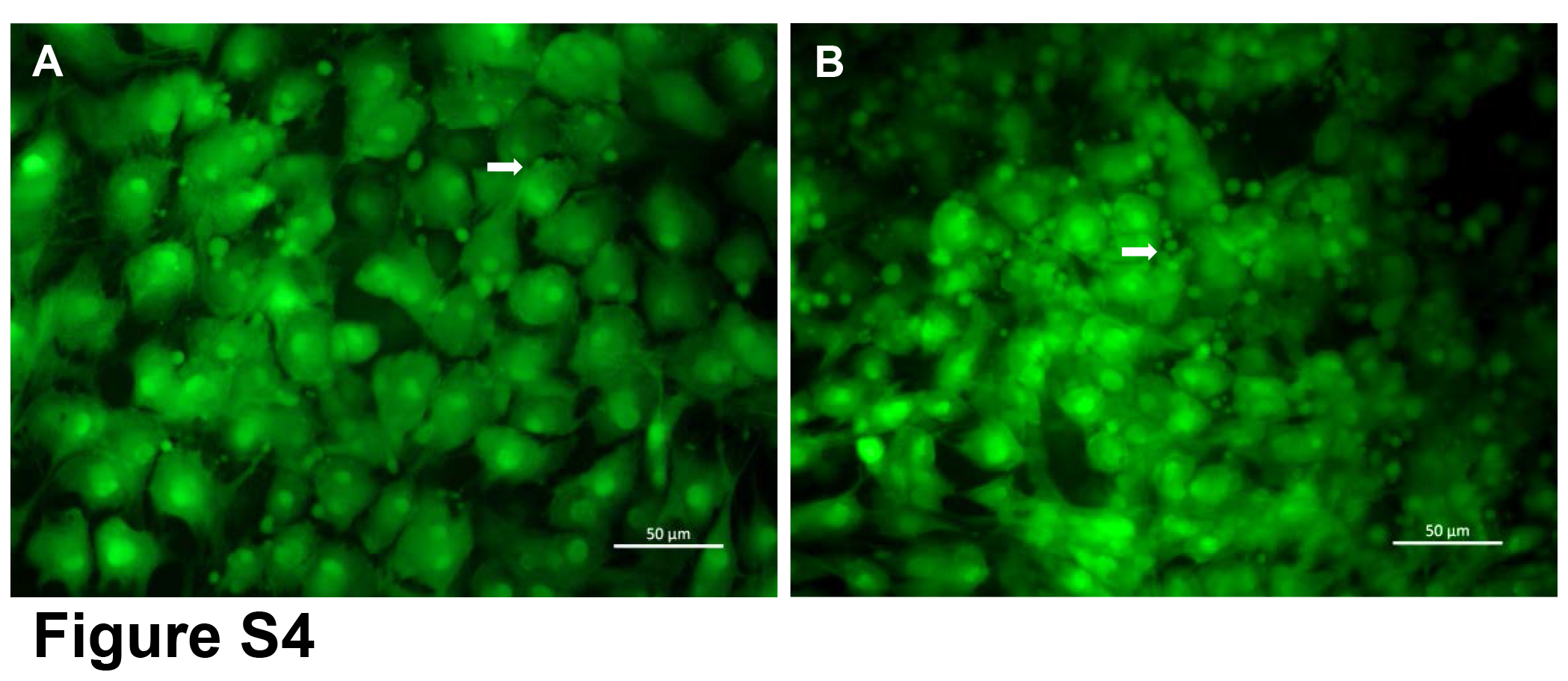

Supplement: Additional file 5: — is Figure S4 showing BC analysis. CDFDA is internalized by hASC-HLCs cultured on collagen type I-coated glass slides (A) and on 2D-AHAM (B), cleaved by intracellular esterases, and excreted into the BC as fluorescent CDF. Arrow shows the location of the fluorescent CDF. Scale bars: 50 μm. (TIFF 5628 kb) [file 13287_2015_208_MOESM5_ESM.tiff]

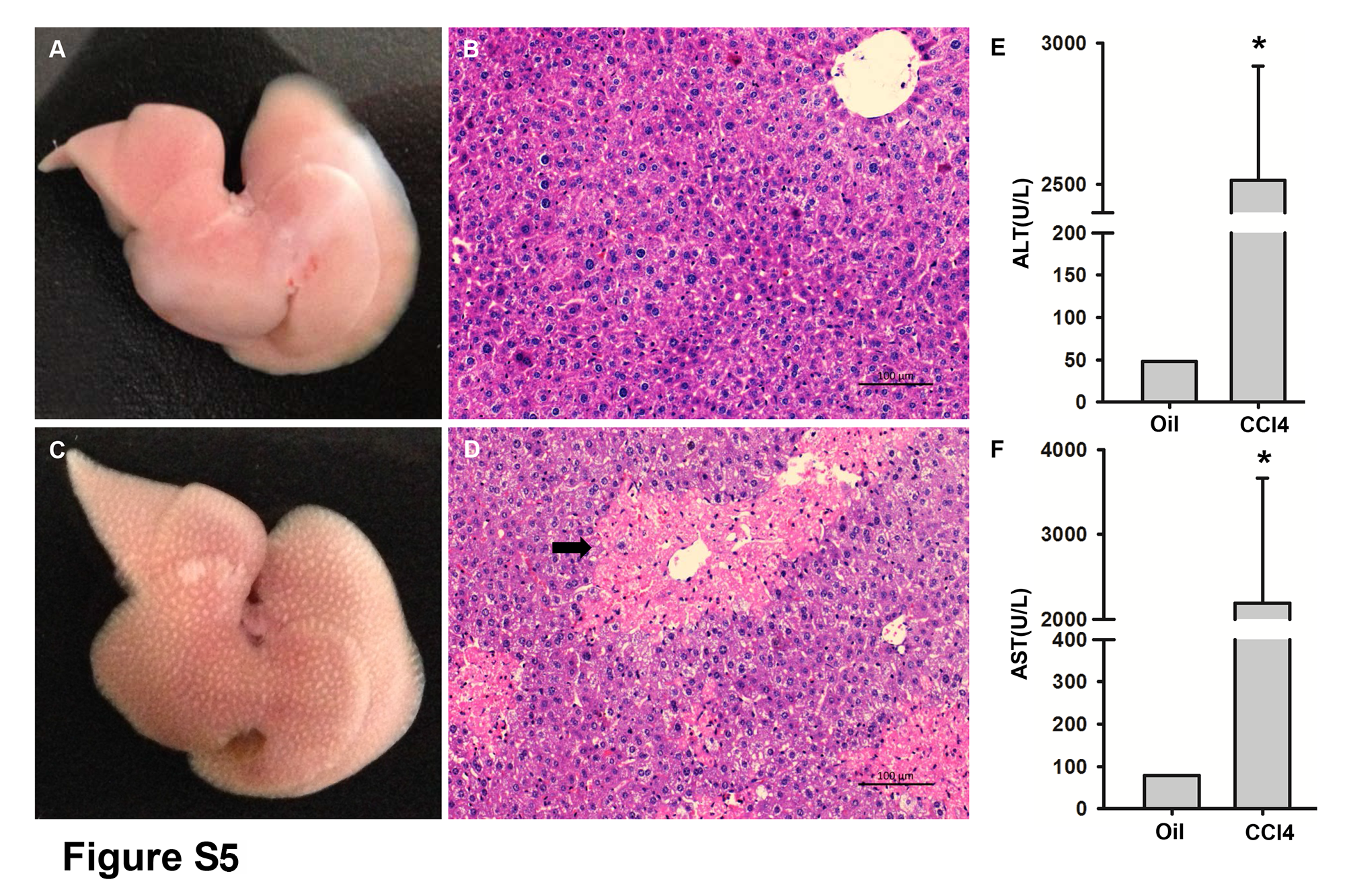

Supplement: Additional file 6: — is Figure S5 showing acute liver injury in BALB/c nude mice induced with CCL4. The general appearance of the liver (A) and H&E staining (B) in BALB/c nude mice that were intraperitoneally injected with olive oil. Gross appearance of the liver (C) and H&E staining (D) in BALB/c nude mice that were intraperitoneally injected with CCL4. At 24 hours post injection, white spots were found at the surface of the liver (C), and H&E staining (D) showed more numerous and larger necrotic areas in the liver around the central venous (black arrow). Scale bar: 100 μm. Plasma levels of ALT (E) and AST (F) were detected in the CCl4-induced acute liver injury group and in the control. *Statistically significant compared to the control group (p <0.05). (TIFF 7839 kb) [file 13287_2015_208_MOESM6_ESM.tiff]

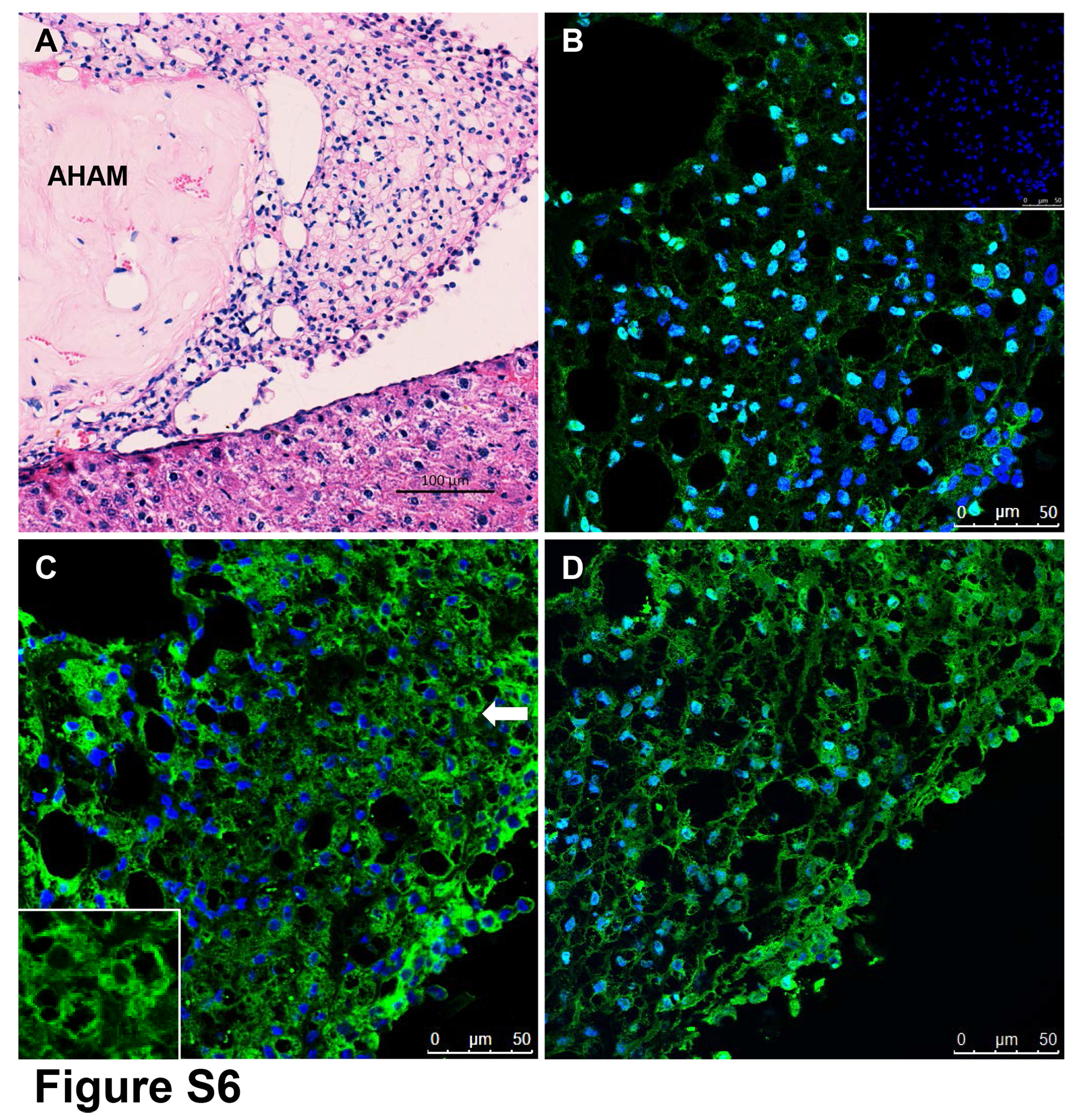

Supplement: Additional file 7: — is Figure S6 showing properties of the hASC-HLC–3D-AHAM graft in the injured liver at day 7 following transplantation. (A) H&E staining analysis of the graft at day 7 post implantation. Scale bar: 100 μm. Immunofluorescence staining of human nuclei (B), ALB (C), and HNF4α (D) in the hASC-HLC–3D-AHAM graft in the CCL4-injured liver at 1 week post implantation. IgG as negative control (B, insert). Scale bar: 50 μm. (TIFF 9840 kb) [file 13287_2015_208_MOESM7_ESM.tiff]

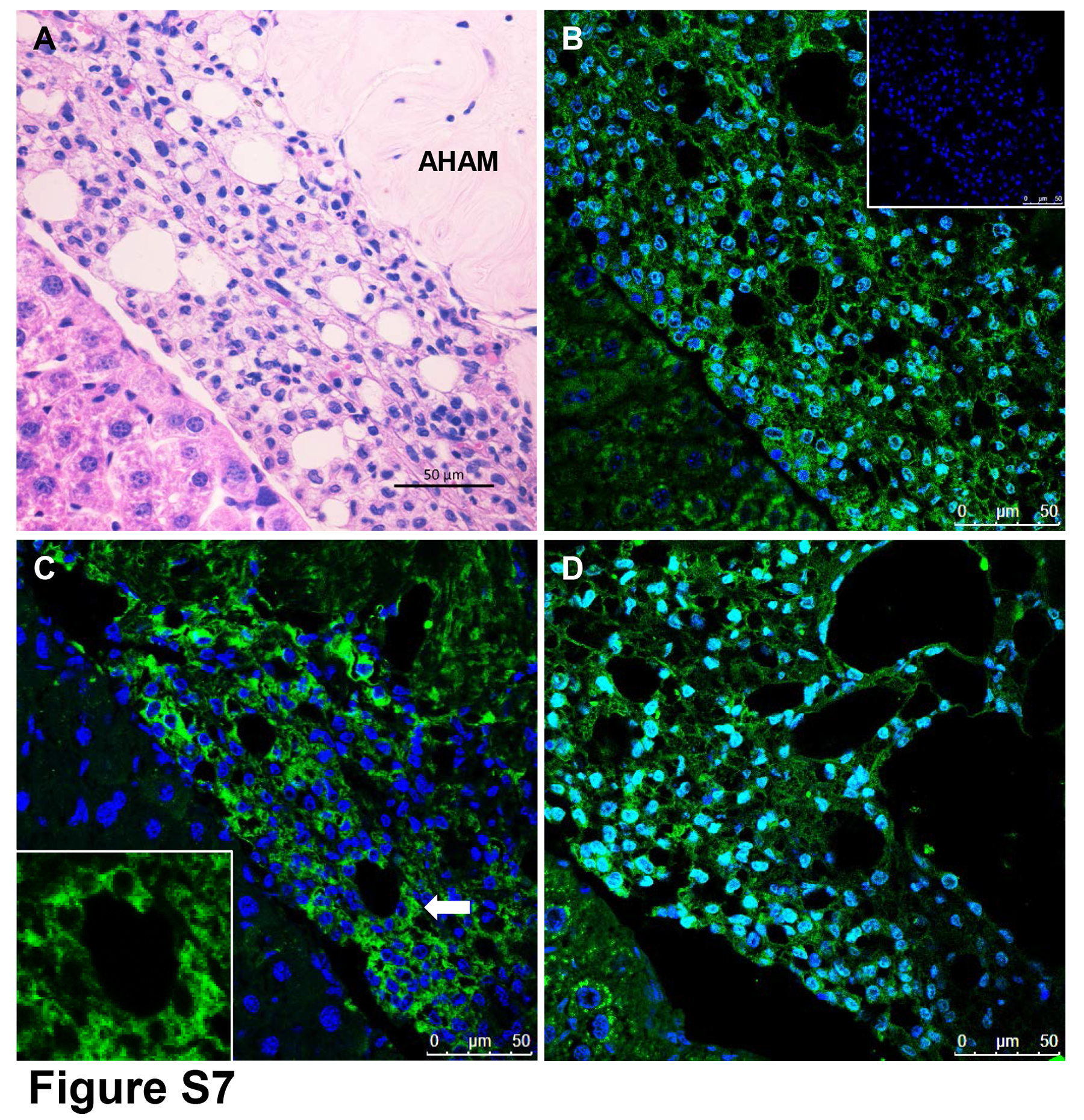

Supplement: Additional file 8: — is Figure S7 showing properties of the hASC-HLC–3D-AHAM graft in the injured liver at day 14 following transplantation. (A) H&E staining analysis of the graft at day 14 post implantation. Immunofluorescence staining of human nuclei (B), ALB (C), and HNF4α (D) in the hASC-HLC–3D-AHAM graft in the CCL4-injured liver at 2 weeks post implantation. IgG as negative control (B, insert). Scale bar: 50 μm. (TIFF 9846 kb) [file 13287_2015_208_MOESM8_ESM.tiff]

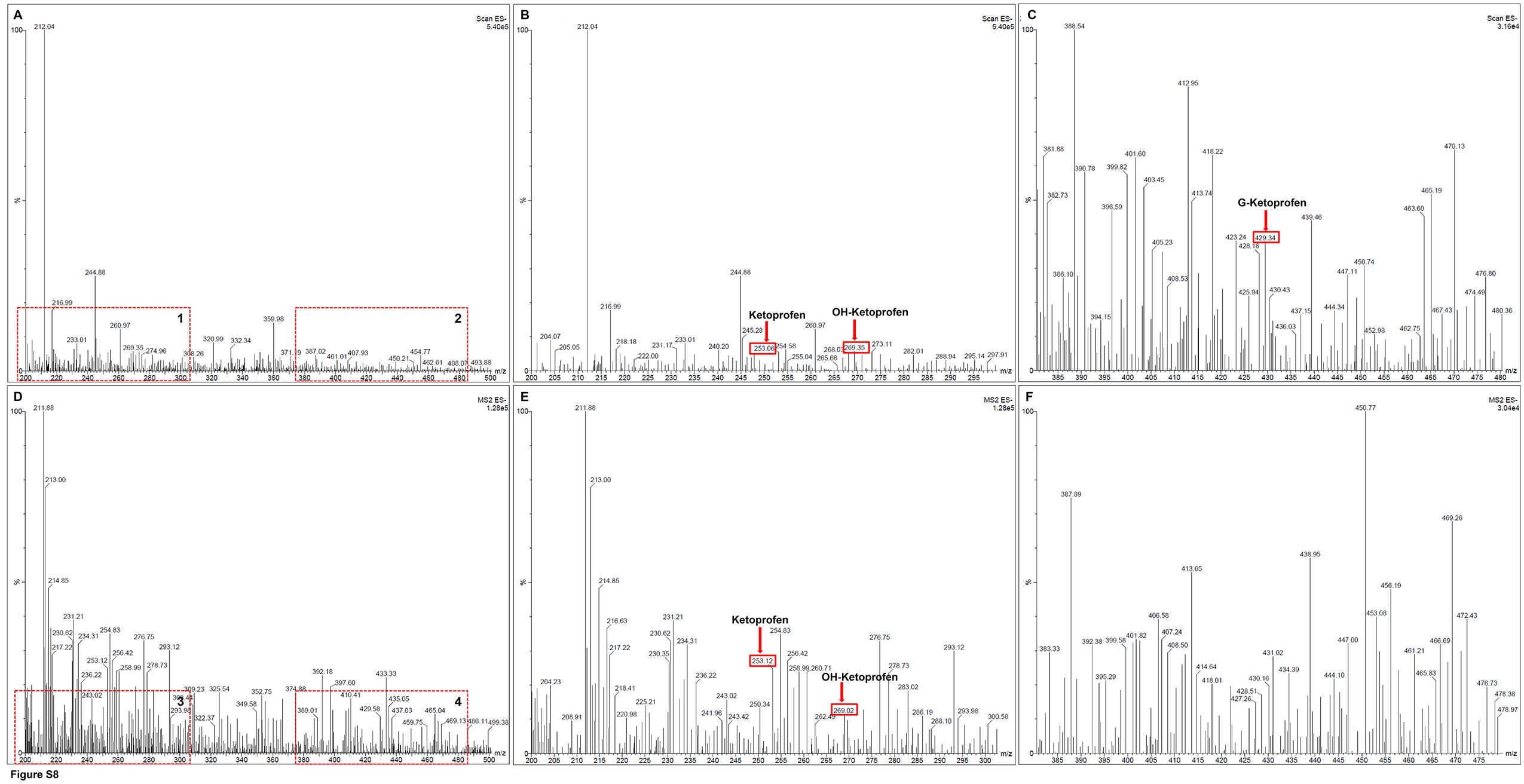

Supplement: Additional file 9: — is Figure S8 showing drug metabolism activity of the hASC-HLC–3D-AHAM graft in the mouse liver at day 56 post implantation. Ketoprofen (15 mg/kg; Sigma-Aldrich) was administrated intravenously to the mice post transplantation of the hASC-HLC–3D-AHAM graft (A, B, C) or 3D-AHAM (D, E, F) in the injured liver. Urine was collected 2 hours after administration. Then 100 μl urine was mixed with 100 μl of 0.5 M acetate buffer (pH 5.0), and then 10 μl of 1 N KOH was added to urine samples, incubated at 80 °C for 3 hours, neutralized by 10 μl of 1 N HCl, and then centrifuged (15,000 rpm, 4 °C, 5 minutes). The supernatant was subjected to mass spectrometry (Quattro micro API; Waters). The ionspray voltage was –4500 V and analyzed m/z transition (Q1/Q3) for ketoprofen,1-hydroxyketoprofen, glucuronide-conjugated ketoprofen was 253.06, 269.35, and 429.34, respectively. (B) Amplification of part 1, (C) amplification of part 2, (E) amplification of part 3, (F) amplification of part 4. Arrow shows the location of the ketoprofen, 1-hydroxyketoprofen, glucuronide-conjugated ketoprofen, OH-ketoprofen: 1-hydroxyketoprofen; G-ketoprofen: glucuronide-conjugated ketoprofen. (TIFF 9829 kb) [file 13287_2015_208_MOESM9_ESM.tiff]
